# Pediatric cardiovascular interventional devices: effect on CMR images at 1.5 and 3 Tesla

**DOI:** 10.1186/1532-429X-15-54

**Published:** 2013-06-19

**Authors:** Sarah N Khan, Stanislas Rapacchi, Daniel S Levi, J Paul Finn

**Affiliations:** 1 Department of Radiological Sciences, David Geffen School of Medicine, University of California at Los Angeles, Peter V. Ueberroth Building Suite 3371, 10945 Le Conte Ave, Los Angeles, CA 90095-7206, USA; 2 Department of Pediatrics, Pediatric Cardiology, Mattel Children Hospital, University of California at Los Angeles, Los Angeles, California, USA

**Keywords:** Cardiovascular, Magnetic resonance, MRA, Pediatric, Stent, Embolization coil, Stainless steel, Nitinol, Artifact, Image quality

## Abstract

**Background:**

To predict the type and extent of CMR artifacts caused by commonly used pediatric trans-catheter devices at 1.5 T and 3 T as an aid to clinical planning and patient screening.

**Methods:**

Eleven commonly used interventional, catheter-based devices including stents, septal occluders, vascular plugs and embolization coils made from either stainless steel or nitinol were evaluated ex-vivo at both 1.5T and 3T. Pulse sequences and protocols commonly used for cardiovascular magnetic resonance (CMR) were evaluated, including 3D high-resolution MR angiography (MRA), time-resolved MRA, 2D balanced-SSFP cine and 2D phase-contrast gradient echo imaging (GRE). We defined the signal void amplification factor (F) as the ratio of signal void dimension to true device dimension. F1 and F2 were measured in the long axis and short axes respectively of the device. We defined F3 as the maximum extent of the off-resonance dark band artifact on SSFP measured in the B_0_direction. The effects of field strength, sequence type, orientation, flip angle and phase encode direction were tested. Clinical CMR images in 3 patients with various indwelling devices were reviewed for correlation with the in-vitro findings.

**Results:**

F1 and F2 were higher (p<0.05) at 3T than at 1.5T for all sequences except 3D-MRA. Stainless steel devices produced greater off-resonance artifact on SSFP compared to nitinol devices (p<0.05). Artifacts were most severe with the stainless steel Flipper detachable embolization coil (Cook Medical, Bloomington, IN), with F1 and F2 10 times greater than with stainless steel stents. The orientation of stents changed the size of off-resonance artifacts by up to two fold. Sequence type did influence the size of signal void or off-resonance artifact (p<0.05). Varying the flip angle and phase encode direction did not affect image artifact.

**Conclusion:**

Stainless steel embolization coils render large zones of anatomy uninterpretable, consistent with predictions based on ex-vivo testing. Most other commonly used devices produce only mild artifact ex-vivo and are compatible with diagnostic quality in-vivo studies. Knowledge of ex-vivo device behavior can help predict the technical success or failure of CMR scans and may preempt the performance of costly, futile studies.

## Background

Trans-catheter procedures in children with congenital heart disease have become a mainstay of therapy for many lesions. These procedures involve the placement of stents, vascular occluders, embolization devices and many other implantable devices. Serial follow up imaging is often performed with cardiovascular magnetic resonance (CMR) to assess the integrity of devices, the patency of blood vessels and stents, and the status of cardiac anatomy and function.

CMR is particularly well suited to patients with congenital heart disease because of the quality of modern imaging, the lack of ionizing radiation, freedom from contrast nephrotoxicity and the likelihood of multiple follow up studies.

CMR and magnetic resonance angiography (MRA) have become the gold standard for imaging many subgroups of congenital heart disease patients, e.g. assessing right ventricular and pulmonary vascular status in treated Tetralogy of Fallot (ToF) patients. However, MR signal loss and off-resonance artifacts from some interventional devices can severely disrupt the signal pattern and render images non-diagnostic. Currently, cardiologists and radiologists are often uncertain how much artifact any given device will create before actually performing the study. Ideally one could make an informed decision about whether or not a study should be undertaken, particularly if anesthesia is required. Moreover, whereas imaging at 3T has potential advantages over 1.5T due to enhanced signal to noise ratio (SNR) [1,2], metal artifacts are potentially more problematic at 3T than at 1.5T [3].

To our knowledge there have been no published reports on the type and extent of MR image artifact caused by pediatric interventional devices.

The goal of our study was to define, by ex-vivo phantom experiments, the influence of magnetic field strength, pulse sequence type, device orientation, flip angle and voxel size on the production of image artifacts from commonly used pediatric, cardiovascular interventional devices.

## Methods

### Experimental setup

Eleven devices, detailed in Table 1 and Table 2, were positioned in a rectangular polypropylene container measuring 22.5 × 14.75 × 5.75 inches, and were tied to an acrylic board (measuring 12 × 12 inches) with polyester thread with spacing of 5 cm in line with ASTM testing standards. The board, placed in the polypropelene container, rested on four equal sized pieces of putty to suspend it above the container floor. An aqueous solution containing 15 ml of Gadobutrol (Bayer-Schering, Germany) (602.74 mg/ml) 10-[(1SR,2RS)-2,3-dihydroxy-1-hydroxy-methylpropyl]-1,4,7,10-tetraazacyclo-dodecane-1,4,7-triacetic acid, gadolinium-complex with 3Liters of normal saline (dilution 1:200) was poured into the container to fully immerse the devices and to provide uniform surrounding magnetic susceptibility. To change the orientation of the devices in the magnetic field, the board was lifted from the container and rotated 90 degrees before being replaced into the container.

**Table 1:** **Device indications**/ **Contraindications**

**Device number**	**Device name**	**Description**	**Indication**	**Contraindication**
1,2	Palmaz Genesis Transhepatic Biliary Stent	Unmounted, balloon-expandable; laser cut stent made from 316L stainless steel tubing.	Palliation of malignant neoplasms in the biliary tree	- Stenting of a perforated duct where leakage from the duct could be exacerbated by the prosthesis;
				- Patients with bleeding disorders or who cannot receive anticoagulants
				- Severe ascites
3	Jostent coronary stent graft	High grade surgical stainless steel (316L) manufactured from a solid tube using precision laser technology adjacent to expandable PTFE graft material.	Free coronary perforations defined as free contrast extravasation into the pericardium, in native coronary vessels or saphenous vein bypass grafts greater than or equal to 2.75mm in diameter	- Bleeding disorders;
				- Inability to take anticoagulants;
				- A lesion which prevents complete inflation of an angioplasty balloon
4,5,6	Intrastent Mega LD and Max LD Biliary stents	Balloon expandable stents made from a stainless steel tube cut into an open lattice.	Palliative treatment of malignant neoplasms in the biliary tree	no known contraindications
7	Amplatzer Vascular Plug	Self expandable nitinol mesh occlusion device, with screw attachment and marker bands at either side.	Arterial and venous embolizations in the peripheral vasculature	no known contraindications
8	Amplatzer Atrial Septal Occluder	Percutaneous, transcatheter, atrial septal defect closure device intended for the occlusion of atrial septal defects (ASD), made of a Nitinol mesh filled with polyester fabric sewn into place by polyester thread.	- Echocardiographic evidence of ASD;	- Intracardiac thrombus
			- Right ventricular volume overload	- Coagulation disorders
			- Closure of the fenestration from a prior fenestrated fontan procedure	- Inadequate vessel size,
				- Thinned septum which will not secure the device
9	Helex Occluder	ePTFE patch material supported by a single nitinol wire frame.	Transcatheter closure of ostium secundum atrial septal defects. Over several months cells being to infiltrate and grow over the ePTFE material for closure of the defect	- Intracardiac thrombi, vasculature which cannot accommodate the introducer sheath,
				- If position required is too close to valves
				- Patients in whom anticoagulation is contraindicated
10	Flipper Detachable Embolization Coil	Stainless steel wire with synthetic fibers,	Arterial and venous embolization of peripheral vasculature	no known contraindications
11	Nit-Occlud Coil	Nitinol coil designed for patent ductus arteriosus (PDA) It has a stiffer aortic side, and a more flexible pulmonary side for this purpose.	Closure of Patent ductus arteriosus (PDA)	no known contraindications

**Table 2:** Device characteristics

**Number**	**Device name**	**Manufacturer**	**Material**	**Length (mm)**	**Diameter (mm)**
1	Palmaz genesis transhepatic biliary stent	Cordis corporation, NJ	stainless steel 316L	22	6
2	Palmaz genesis transhepatic biliary stent	Cordis corporation, NJ	stainless steel 316L	14	5
3	Jostent coronary stent	Abbott Vascular, CA	stainless steel 316L +ePTFE	16	3
4	Intrastent LD max biliary stent	EV3, Inc. MN	stainless steel 316L	26	12
5	Mega LD biliary stent	EV3 Inc. MN	stainless steel 316L	26	10
6	Mega LD biliary stent	EV3 Inc. MN	stainless steel 316L	36	12
7	Amplatzer vascular plug (IV)	AGA Medical Corporation, MN	nitinol mesh + platinum marker bands		20
8	Amplatzer atrial septal occluder	AGA Medical Corporation, MN	nitinol + polyester		29
9	Helex septal occluder	Gore Medical, AZ	nitinol frame + ePTFE	7 (height)	25
10	Flipper detachable embolization coil	Cook Medical, IN	stainless steel with synthetic fibers	3 (height)	5
11	Nit-occlud PDA occlusion coil	PFM Medical Inc, CA	nitinol	6 (height)	12

### Static magnetic field strength

The experiments were conducted at 3T and at 1.5T. The 3T system was a 32 channel whole body scanner (Magnetom TIM Trio, Siemens Medical Solutions, Malvern, PA) with the following specifications: gradient strength 40 mT/m, slew rate 200 mT/m/ms, bore diameter 60 cm. The 1.5T system was a 32 channel whole body scanner (Magnetom TIM Avanto, Siemens Medical Solutions, Malvern, PA), with the following specifications: gradient strength 45 mT/m, slew rate 200 mT/m/ms, bore diameter 60 cm. Both scanners were outfitted with similar phased array surface coils and spine coils and operated at the same software level.

### Pulse sequence type

Imaging was performed with sequences routinely employed for cardiovascular imaging at our institution, including: 2D balanced-Steady State Free Precession (SSFP) for cardiac cine imaging and 2D-flow encoded Gradient Echo for phase-contrast flow measurements, high-resolution, spoiled 3D Gradient Echo (GRE) for contrast enhanced MRA and spoiled 3D GRE with view-sharing for time-resolved contrast enhanced MRA (TWIST).Sequence parameters are summarized in Table 3. Sequence parameters reflected those employed in clinical protocols. Most parameters were maintained identical between 1.5 T and 3 T, only the flip angle was adapted for 3D sequences to maintain SAR dose compliance. The 2D-SSFP sequence required also the slice thickness, bandwidth and TE/TR to be optimized for each field strength, as is the case with clinical imaging.

**Table 3:** Technical parameters

	**2D balanced-SSFP dynamic 2D balanced-SSFP cine -CMR**		**3D-GRE dynamic time-resolved MRA**		**Phase-contrast 2D-GRE**		**3D-GRE high-resolution MRA**	
	**1**.**5T**	**3T**	**1**.**5T**	**3T**	**1**.**5T**	**3T**	**1**.**5T**	**3T**
TR	4.0	3.61	3.05	3.05	9.4	9.4	3.18	3.18
TE	1.73	1.52	1.28	1.28	2	2	1.31	1.31
Flip angle	60	80	22	25	30	30	19	30
Pixel bandwidth	914	977	751	751	554	554	610	610
Matrix	304×243	320×240	512×347	512×347	192×192	192×192	512×358	512×358
In-plane resolution (mm^2^)	1 × 1.25	1 x 1.33	0.61 × 0.93	0.61 x 0.93	1.66 × 1.66	1.66 × 1.66	0.68 × 0.98	0.68 × 0.98
Slice thickness	2	2.5	1	1	5.5	5.5	1	1
Grappa acceleration factor	2	2	2	2	2	2	2	2

Signal voids are expected in GRE sequences because the metallic devices distort the local magnetic field and cause intra-voxel dephasing. Additionally the SSFP sequence is sensitive to off-resonance effects, and banding artifacts are expected to occur due to the deformation of the magnetic field around the devices.

### Orientation in magnetic field

The effect of varying the orientation of the stents relative to the B0axis was investigated at 1.5T and 3T. For orientation 1, the acrylic board to which the stents were attached was placed in the scanner with the long axis parallel to B0. In orientation 2, the acrylic board was rotated 90 degrees clockwise and the scans were repeated. For analysis purposes, in orientation 1 the ratio of F3 to true long axis length was derived, for orientation 2 the ratio of F3 to true short axis length was derived.

### Flip angle

3D-GRE high-resolution MRA sequences were performed at 1.5T with three flip angles: 15 degrees, 30 degrees and 45 degrees.

### Orientation of phase encode direction

Two directions for phase encoding were tested for each sequence at 1.5T. The size of the F1 and F2 were measured in direction 1 (head-foot direction), and direction 2 (left-right direction).

### Device composition

F1, F2 and F3 were compared for the devices composed of stainless steel and nitinol at 1.5T and 3T to investigate the effect of device composition.

**Figure 1:**
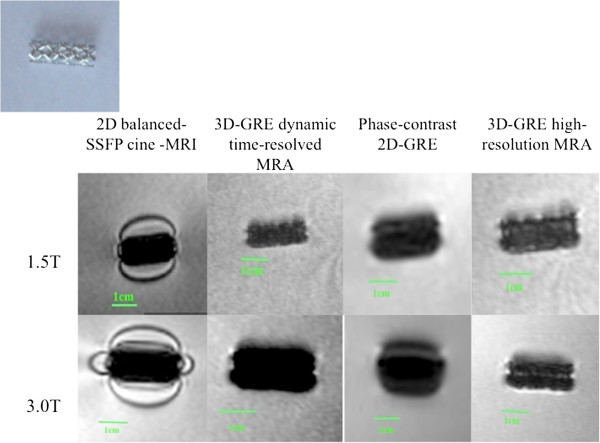
MR appearance of Palmaz Genesis Transhepatic Biliary stent (Device 1,2).

**Figure 2:**
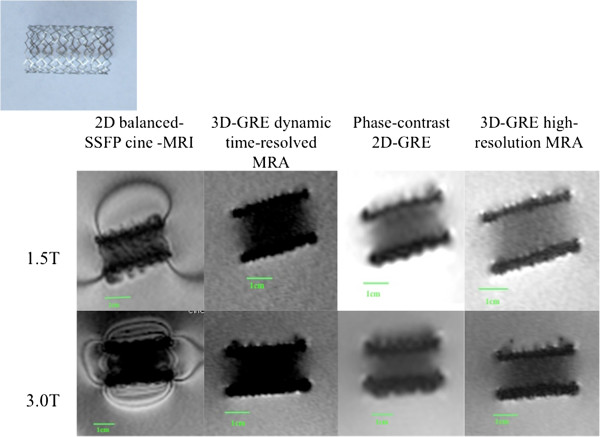
MR appearance of EV3 Transhepatic Biliary Stent (Devices 4-6).

**Figure 3:**
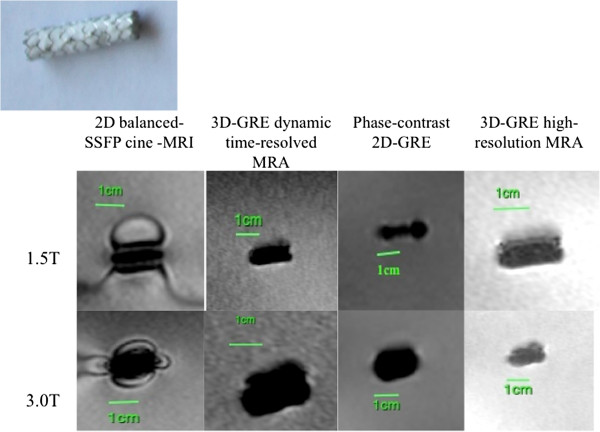
MR appearance of Jostent Coronary Stent Graft (Device 3).

**Figure 4:**
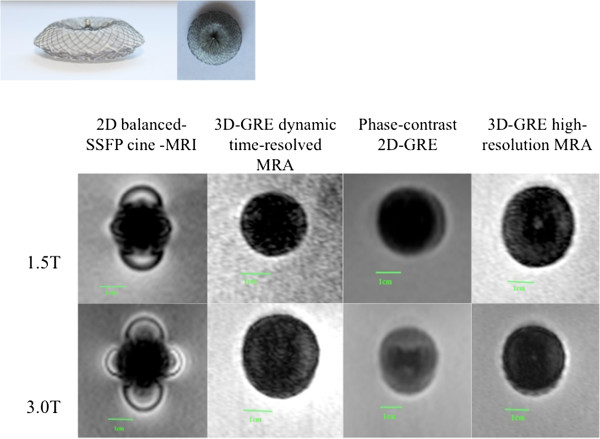
MR appearance Atrial Septal Occluder (Device 7).

**Figure 5:**
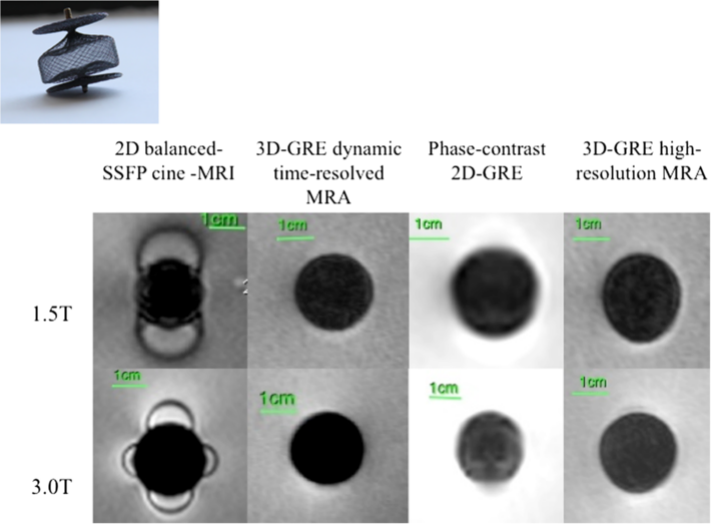
MR appearance of Amplatzer Vascular Plug (Device 9).

**Figure 6:**
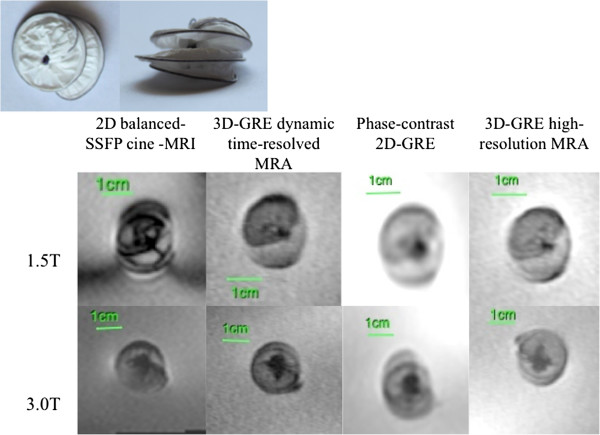
MR appearance of Helex Occluder (Device 8).

**Figure 7:**
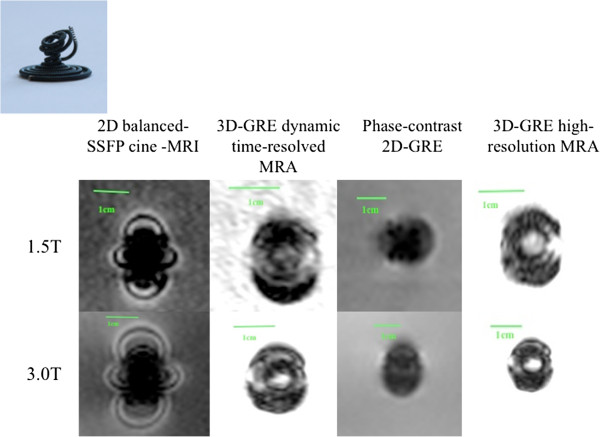
MR appearance of Nitocclud Embolization Coil (Device 10).

**Figure 8:**
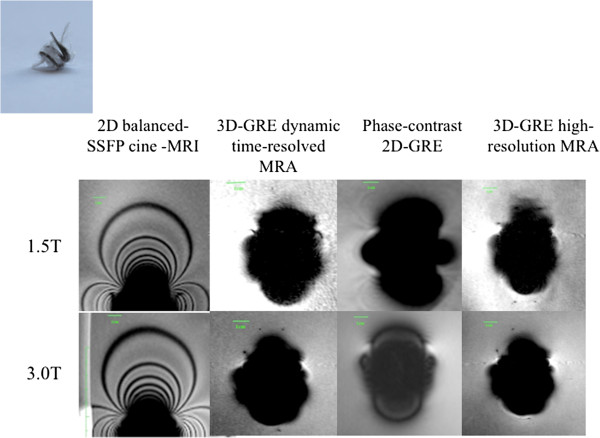
MR appearance of Flipper Detachable Embolization coil (Device 11).

All six stents (see examples in Figures 1 and 2) studied were composed of stainless steel (316L). The Jostent coronary stent (Abbott Vascular, CA) also contains a ePTFE covering (Figure 3). The Amplatzer septal occluder (AGA Medical Corporation, MN) is composed of nitinol and polyester (Figure 4), the Amplatzer vascular plug (AGA Medical Corporation, MN) has a nitinol mesh and platinum marker bands (Figure 5). The Helex septal occluder (Gore Medical, AZ) is composed of nitinol and ePTFE (Figure 6). The Nit-occlud coil (PFM Medical Inc, CA) is made solely of nitinol (Figure 7), the Flipper detachable embolization coil (Cook Medical, IN) is composed of stainless steel within synthetic fibers (Figure 8).

### Artifact assessment

For quantitative analysis of the artifacts, the following measurements were made:

1. The signal void amplification factor (F) was defined as the ratio of signal void dimension to true device dimension.

2. F1 and F2 were measured in the long axis and short axes respective to the device.

3. F3 was defined as the maximum dimension of the off-resonance band artifact on SSFP (measured in the B0 direction).

### Statistical analysis

Average and standard deviation values were calculated for F1, F2 and F3. The one way ANOVA test was used to compare F1 values between the four pulse sequences at 1.5T and 3T. A paired samples t-test was used to determine significant differences between 1.5T and 3T for F1, F2 and F3 values. An independent samples t-test was used to determine significant difference between stainless steel and nitinol composition.

### Clinical correlation

The CMR studies of three patients with congenital heart disease treated by coil embolization (2 patients) or an Amplatzer occluder device were reviewed, for correlation with the ex-vivo findings of the same devices.

## Results

### Flipper detachable embolization coil

**Figure 9:**
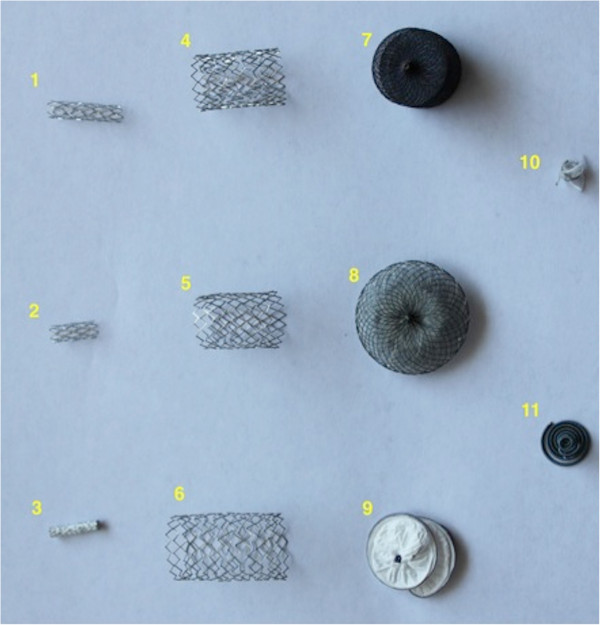
Photographic representation of devices.

The Flipper detachable embolization coil (Cook Medical, Bloomington, IN) (see Figure 9, Device 10) exhibited substantially greater magnetic properties than the other 10 devices and produced much larger F1, F2 and F3 than the other devices. The F1 and F2 measurements for each sequence were: 2D balanced-SSFP cine (7.2, 5.3), time-resolved MRA (7.2, 5.1), phase-contrast 2D-GRE (8.1, 5.8) and 3D high-resolution MRA (6.6, 5.4). The F3 for 2D balanced-SSFP was 19.2.

### Static magnetic field strength

Mean and standard deviation values for F1 and F2 significantly lower for at 1.5T than 3T for the 2D balanced SSFP cine sequence, time-resolved MRA sequence and phase contrast 2D-GRE sequence, but not for the 3D-GRE high-resolution MRA sequence (refer to Table 4).

**Table 4:** **Amplification factors F1 and F2 for all tested sequences at 1**.**5T and 3T**

		**1**.**5T**	**3T**	**p value**
2D balanced SSFP cine	F1	1.1± 0.24	1.2 ± 0.21	0.002
	F2	1.9± 1.00	2.1 ± 1.02	0.040
3D-GRE dynamic time-resolved MRA	F1	1.1± 0.20	1.2 ± 0.16	0.017
	F2	1.5± 0.52	1.8 ± 0.81	0.022
Phase-contrast 2D-GRE	F1	1.2± 0.19	1.3 ± 0.25	0.035
	F2	2.1± 1.06	2.5 ± 1.37	0.005
3D-GRE high-resolution MRA	F1	1.1± 0.15	1.1 ± 0.15	0.741
	F2	1.6± 0.49	1.7 ± 0.73	0.249

### Pulse sequence type

There was a significant difference in F1 between the individual pulse sequences at 1.5T (2D balanced-SSFP cine 1.1 ± 0.24; time-resolved MRA 1.1 ± 0.20; phase-contrast 2D-GRE 1.2 ± 0.19; 3D-GRE high-resolution MRA 1.1 ± 0.15; p = 0.010)and 3T (2D balanced-SSFP cine 1.2 ± 0.21; time-resolved MRA 1.1 ± 0.16; phase-contrast 2D-GRE 1.3 ± 0.25; 3D-GRE high-resolution MRA 1.1 ± 0.15; p = 0.011).

### Orientation in magnetic field

At 1.5T, rotation of the acrylic board from orientation 1 (long axis of stent parallel to B0) to orientation 2 (long axis of stent perpendicular to B0) resulted in an increase in off resonance artifact by factor of 1.3 - 2.5 for stents 1,2,4,5,6. At 3T, rotation of the acrylic board resulted in an increase in off resonance artifact by a factor of 0.8-1.7 for stents 1,2,4,5,6. Stent 3 demonstrated an increase in off resonance artifact by a factor of 3 from orientation 1 to orientation 2 at 1.5T and 3T.

### Flip angle

There was no significant difference (p>0.05) in F1 and F2 values for different flip angles of 15 degrees,30 degrees and 45 degrees F1(2.9 ± 1.29; 2.9 ± 1.40; 2.9 ± 1.36; p = 0.262) and F2 (1.9 ± 1.22; 1.9 ± 1.31; 1.9 ± 1.27; p = 0.899).

### Orientation of phase encode direction

There was no significant difference between phase encode direction 1 and direction 2 for any of the four tested sequences (refer to Table 5).

**Table 5:** **Amplification factors F1 and F2 for phase encode direction 1 and 2 for all tested sequences** (**p** >**0**.**05**)

		**Phase encode direction 1**	**Phase encode direction 2**	**P value**
2D balanced SSFP cine	F1	2.6 ± 0.63	2.7 ± 0.58	0.401
	F2	1.6 ± 0.77	1.7 ± 0.88	0.067
	F3	4.0 ± 1.05	4.3 ± 1.00	0.256
3D-GRE dynamic time-resolved MRA	F1	2.9 ± 1.40	2.9 ± 1.42	0.251
	F2	1.9 ± 1.32	2.0 ± 1.30	0.706
Phase-contrast 2D-GRE	F1	3.3 ± 1.66	3.4 ± 1.80	0.936
	F2	2.3 ± 1.48	2.4 ± 1.65	0.079
3D-GRE high-resolution MRA	F1	2.9 ± 1.28	2.9 ± 1.47	0.483
	F2	2.0 ± 1.24	2.1 ± 1.77	0.132

### Device composition

F3 was significantly greater for stainless steel stents than nitinol stents at both 1.5T (stainless steel 5.7 ± 1.53; nitinol 2.2 ± 1.01; p= 0.001) and 3T (stainless steel 5.9 ± 1.34; nitinol 1.9 ± 0.93; p=0.002). No significant difference (p> 0.05) was found in F1 and F2 for stainless steel and nitinol stents: 1.5T (stainless steel 1.1 ± 0.14; nitinol 1.1 ± 0.26) and 3T (stainless steel 1.2 ± 0.12; nitinol 1.2 ± 0.28).

## Discussion and conclusion

In our study, the stainless steel Flipper detachable embolization coil (Cook Medical, Bloomington, IN) caused significant disruption to images. The majority of other transcatheter devices did not cause significant signal void, with minimal signal disruption beyond the immediate vicinity of the device, regardless of field strength or pulse sequence type. We recommend careful consideration of whether an MR study should be performed in a child with such a coil in place, if the anatomy of interest is within 5 cm of this device.

The results of our study suggest the strength of the magnetic field, device composition, sequence type and orientation of the device in B0 have the largest impact on the amplification factor of implanted devices in an MR environment. The flip angle and direction of the phase encode direction do not affect amplification. Certain devices, such as the stainless steel embolization coil cause significant disruption of images, and patients with such devices should undergo careful consideration before scans are performed. The prior knowledge of artifact size and proximity to an anatomical structure of interest may influence the decision to perform a CMR study, or allow the study to be planned with optimal parameters to minimize artifacts. In children who often require general anesthesia for an, the risks of anesthesia may not outweigh the benefit of the scan if there is considerable image disruption. This knowledge could also influence the choice of device by the interventional pediatric cardiologist.

Larger artifact size was encountered at 3T than 1.5T. Imaging at higher magnetic field strength results in stronger susceptibility artifacts [4,5]. In a study comparing artificial lumen narrowing in stents of stainless steel, nitinol and cobalt alloy with 3D-GRE high-resolution MRA images at 3T and 1.5T, the largest amount of narrowing was found in stainless steel and cobalt alloy stents at 1.5T and the least narrowing was detected in nitinol stents at 3T [6].

Our results found stainless steel devices to produce more artifact than nitinol devices. The 316 low carbon alloy of stainless steel is austenitic and non-magnetic [7], however manufacture of the complex shape of a stent can produce ferromagnetism in the stent. Nitinol is a nickel-titanium shape memory alloy. It is well suited to stent composition as it is biologically inert, has elastic properties, is non-thrombogenic and resistant to calcification and erosion [8]. Several authors [4,9-11] have found improved MR compatibility from nitinol devices compared to stainless steel. Holton et al, supported these findings with isolating only the compositional contribution to signal artifact, after normalization of the contribution of geometry-related RF shielding [12]. In another study comparing the luminal patency and stent induced artifacts for platinum based stents with stainless steel, nitinol, cobalt alloy and tantalum stents, only platinum based stents showed less than 30% luminal narrowing due to artifact [13].

While the ferromagnetic properties of a device correlate with the expected size of image artifacts, non-ferromagnetic devices can also produce significant image artifacts. This is thought to be due to a reduction of radiofrequency amplitude near the device which depends on its shape and is most pronounced near edges and points of the metallic surface [14].

In our study, increased artifact was found when stents were placed with the long axis perpendicular to B0. Hug *et al*, also found largest artifact is observed when the position of stents was perpendicular to B0, smallest artifact occurs when stent is parallel to B0 [15]. Parallel orientation of stents in the magnetic field has been shown to increase lumen visibility [4].

Our findings suggest a significant difference in artifact size between difference imaging sequences. In another study of 15 coronary artery stents deployed in 15 healthy swine coronary arteries, vessel visualization and image artifact was compared between a 3D cartesian gradient echo sequence, a 3D spiral gradient echo sequence and a cardiac triggered 3D SSFP sequence [16]. With these three sequences, no difference in SNR was found inside or outside the stent. With 3D SSFP sequence however, a smaller vessel diameter was found compared to the 3D spiral gradient echo and 3D Cartesian gradient echo sequences. However, other investigators have reported SSFP sequences to be less sensitive to susceptibility artifact [17,18].

An increase of the flip angle results in higher radiofrequency power of the excitation pulse and improved lumen visualization [19,20]. Holten *et al* investigated the optimal flip angle at 1.5T for minimal reduction of signal inside 3 stents made from: 316L stainless steel, nitinol and ABI alloy. Highest signal for nitinol and stainless steel was found at flip angle of 90 degrees, and for ABI alloy was 270 degrees [21]. Our results did not demonstrate an increase in artifact size at the three tested flip angles (15, 30, 45), perhaps as we only tested a narrow range compared to other authors.

We did not find a significant difference in the size of artifacts encountered between changing the phase encode directions for any sequence. Acquiring the scan with the read direction along static magnetic field results in better lumen visualization [4]. The orientation of the major extent of artifact of ferromagnetic devices is parallel to the frequency encode direction [22].

The Flipper Detachable Embolization Coil (Cook Medical, Bloomington, IN) was found by visual assessment to have distinctly different magnetic properties to the other devices. Cook Medical has labeled stainless steel embolization coils as MR conditional. MR conditional is described as an item has been demonstrated to pose no known hazards in a specified MR environment with specified conditions of use [23]. The newer MReye Flipper Detachable Embolization coil (Cook Medical, Bloomington, IN) is made from Inconel (a nickel-chromium-based superalloy), and acknowledgement is made of the compromise in image quality in or nearby to the position of the coil. The reason behind the vast difference between artifact size for a stainless steel embolization coil and stainless steel stent is unknown, however could be due to the tightly coiled configuration of the wire. The Nit-occlud device is an embolization coil made from nitinol, however this did not demonstrate any significantly large artifacts.

**Figure 10:**
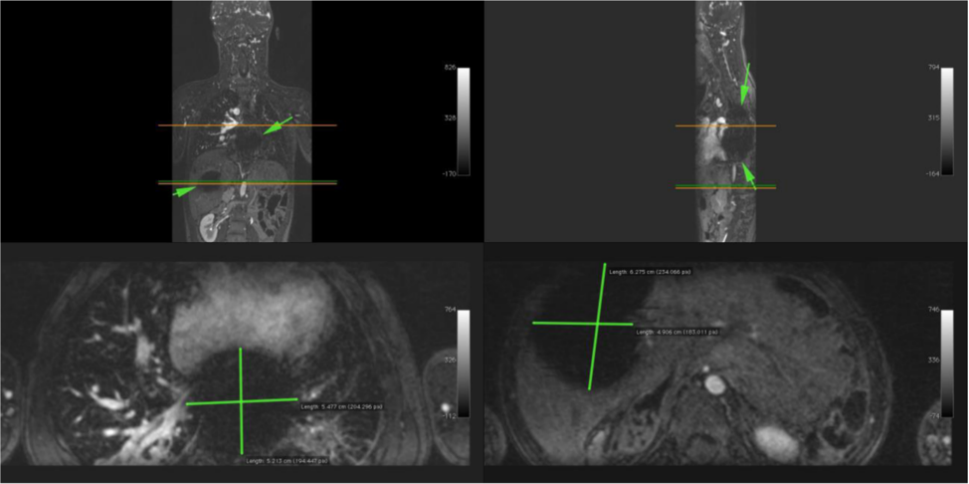
**Image artifact due to Flipper detachable embolization coils in a 5 year old male with heterotaxy, transposition of the great arteries, hypoplastic left ventricle, double outlet right ventricle, bilateral superior vena cavae and hyposplenia with prior history of multiple embolizations.** Contrast enhanced MR angiography was performed at 3T. Reconstructions in the coronal, sagittal and axial planes show extensive areas of signal void, up to 10cm in size, from embolization coils in the lower posterior mediastinum and right hepatic lobe (green arrows).

**Figure 11:**
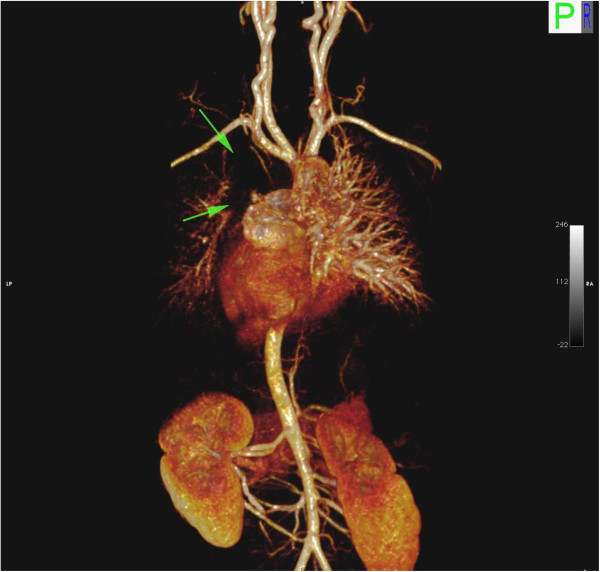
**Image artifact due to Flipper detachable embolization coils in a 5 year old male with heterotaxy, transposition of the great arteries, hypoplastic left ventricle, double outlet right ventricle, bilateral superior vena cavae and hyposplenia with prior history of multiple embolizations.** 3D volume rendered reconstruction from the same study, posterior. Arrows highlight extensive signal void in the left hemithorax.

**Figure 12:**
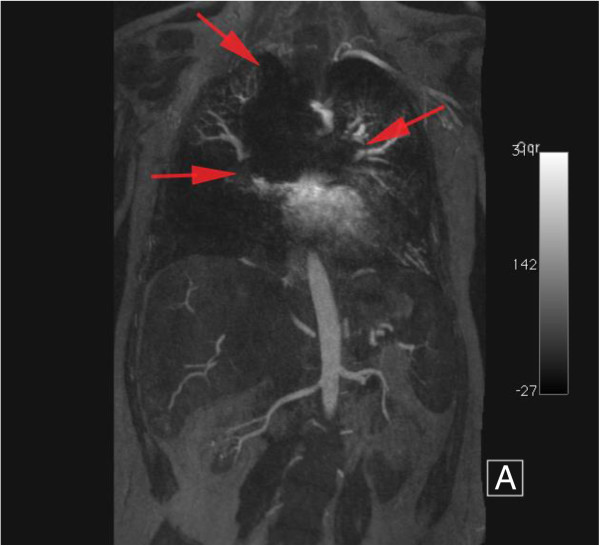
**Image artifact due to Flipper detachable embolization coils in a 5 year old male with heterotaxy, transposition of the great arteries, hypoplastic left ventricle, double outlet right ventricle, bilateral superior vena cavae and hyposplenia with prior history of multiple embolizations.** Maximum intensity projection (MIP) in the coronal plane shows extensive areas of signal void (red arrows).

**Figure 13:**
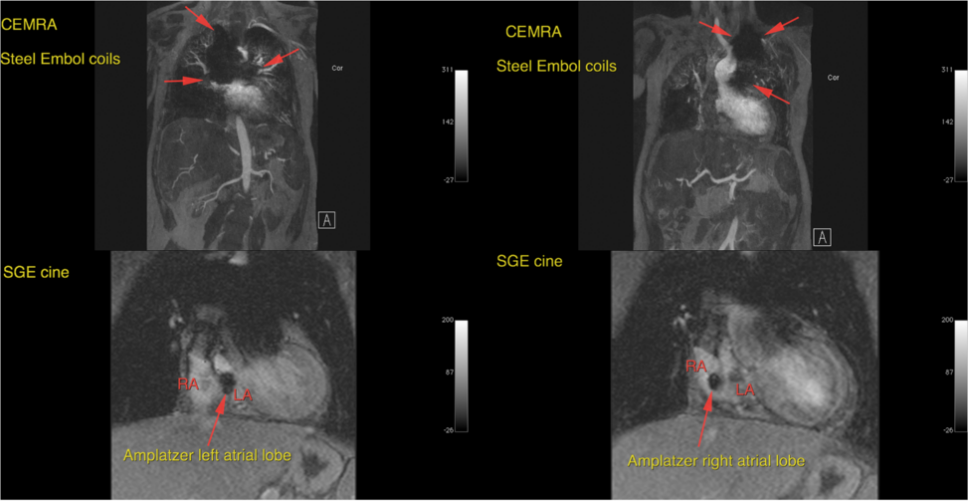
**24 year old female with tricuspid atresia and history of lateral tunnel Fontan procedure.** Contrast enhanced MRA performed at 1.5T (upper images) maximum intensity projections, coronal plane, arterial phase, extensive signal void due to prior placement of Flipper detachable embolization coils is visible. Also SGE cine images demonstrate local signal void from amplatzer occluder device (lower images).

**Figure 14:**
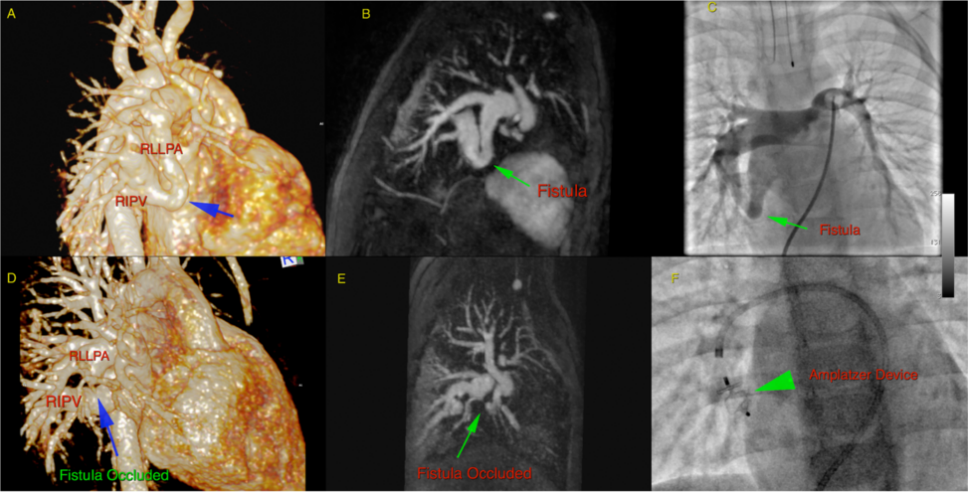
**20 month old infant prior to (upper row) and following** (**lower row**) **occlusion of a right lower lobe pulmonary arteriovenous fistula with an Amplatzer device. **Contrast enhanced MRA performed at 3T: shown are 3D volume rendered reconstructions (**A**,**D**), maximum intensity projections, sagittal plane (**B**,**E**), and procedural angiographic images (**C**,**F**). Images (**A**-**C**) are prior to embolization, (**D**-**F**) post embolization. Only minimal signal void is present and occlusion of the arteriovenous shunt is unequivocally confirmed.

One major limitation to our study was that we were not able to test every parameter for every sequence at both field strengths. Additionally, the range of flip angles we tested was relatively narrow compared to other authors, which led to different conclusions to other published work. However, our study was designed to reflect typical clinical imaging scenarios, rather than to explore a wide extent of parameter space. Secondly, our study did not include any in vivo data, and we did not place the devices in artificial tubes to mimic blood vessels. However we are able to demonstrate the appearance of signal void by various devices seen in patients at our institution (Figures 10,11,12,13,14). Thirdly, only one observer measured F1, F2 and F3, ideally with a second observer we would have evidence of reproducibility to support our findings.

In conclusion, careful consideration of prior implanted devices in a patient is necessary before CMR is performed. Selection of magnetic field strength, sequence type, device composition and orientation in the magnetic field will minimize image disruption from these devices. Stainless steel embolization devices will produce large artifact, whereas stainless steel stents cause minimal disruption.

## Competing interests

The authors have no financial conflicts of interest to report.

## Authors’ contributions

Guarantor of integrity of entire study (PF), Study concepts (PF), Study design (SK), Definition of intellectual content (DL), Literature research (SK), Clinical studies (PF), Experimental studies (SR), Data acquisition (SK/SR), Data analysis (SK), Statistical analysis (SK and SR), Manuscript preparation (SK), Manuscript editing (DL and PF), Manuscript review (SK, SR, DL, PF). All authors read and approved the final manuscript.
